# Supplementary material for: Between a rock and a hard place: experimental assessment of recruitment patterns in a bathyal environment of the Low Arctic
Source: PeerJ. 2025 Aug 22;13:e19850. doi: 10.7717/peerj.19850 (PMC12377359; doi:10.7717/peerj.19850)
Supplement: Supplemental Information 1 [file peerj-13-19850-s001.pdf]

Supplementary material for:

Between a rock and a hard place: experimental assessment of recruitment patterns in a bathyal environment of the Low Arctic

Sophie Wolvin, Jean-François Hamel, Annie Mercier

Supplementary results

Surface location and microhabitat colonization pattern detail

Three colonial hydrozoans (Campanulariidae msp. 3, msp. 2, and *Eudendrium* msp. 1) colonized almost all locations and microhabitats available on the substrata (Figure 6A). The most abundant and opportunistic colonizer (Campanulariidae msp. 3) did not display location preferences, except for corners of stone and wood at Site 2. In terms of microhabitat colonization, colonies extended through most microhabitats available, except into the pores of stone at Site 2 or below the outermost surface of mesh at Site 3. Colonies also extended stolons internally in mesh (Sites 1, 2 and 3). Low colonization was observed below the outermost surface of plastic at Site 4. Campanulariidae msp. 2 consistently colonized corners, edges, and centre of the blocks except for edges and corners on mesh at Site 3. Microhabitats were almost all colonized, except below the outermost surface on mesh at Site 3. *Eudendrium* msp. 1 colonized the corner, edge, and centre locations with no exceptions; all microhabitats available were colonized.

An additional four morphospecies exhibited broad colonization of locations and microhabitats across multiple substratum types and sites (Figure 6A). The colonial cnidarian Octocorallia msp. 1 had variable settling locations across geographic site and substratum type but occurred in one location per block; the exception was broader colonization of mesh at Site 4 where the morphospecies occurred in two recruitment locations. It colonized the outer microhabitat only at Site 1 across three substratum types (mesh, plastic, and stone) and only the inner microhabitat on plastic at Site 4, while again exhibiting broader colonization on mesh as well as stone, where it colonized all microhabitats available. The benthic Foraminifera msp. 1 also occurred in multiple locations on mesh at Site 1 (3 locations), mesh at Sites 2 and 3 (2

locations each), and stone at Site 4 (2 locations). At Site 1 it also occurred on stone and wood in the centre, and at Site 4 on mesh in the centre. Microhabitat colonization was variable: it occurred in the outer microhabitats of mesh, stone, and wood at Site 1 as well as mesh at Sites 2 and 3, while more broadly colonizing most available microhabitats on mesh and stone at Site 4. The only exception was internal occurrence on mesh at Site 1 and 3. The benthic radiolarian Radiolaria msp. 1 conversely was limited to one location per substratum type: it occurred just in the centre of mesh and stone at Site 2 as well as plastic and stone at Site 4. At Sites 3 and 4 it occurred in two microhabitats, inner and internally, while all other occurrences it was in just one microhabitat per substratum type. It was in the middle microhabitat of mesh at Site 2, the outer of plastic at Site 4, and the inner microhabitats of stone at both Site 2 and 4. Individual eggs (Unknown msp. 1) occurred in the centre of plastic at Site 1 and stone at Site 2 as well on the edges of stone at Site 4, while showing up in all locations of stone at Site 1. Its microhabitat colonization was limited, as it occurred in the outer microhabitat of plastic at Site 1, and the inner of stone at Sites 1 and 2.

Six mspp recruited to or colonized few locations and microhabitats across two sites (Figure 6B). The unitary cnidarian Actiniaria msp. 1 had no specific location trend but occurred in one location per substratum type; it was present in the centre of stone at Site 1 and in the centre of plastic at Site 4. Similarly, it occurred in one microhabitat per substratum type: internally on mesh and outer on stone at Site 1, and middle on plastic at Site 4. The colonial hydrozoan Campanulariidae msp. 4 colonized the centre of mesh at Site 1 but more broadly including the edges and corners of mesh at Site 4. Its microhabitat colonization followed a similar pattern, occurring in the outer microhabitat on mesh at Site 1 and all available microhabitats on mesh at Site 4 including extending stolons from the colony internally. Porifera

msp. 1 occurred in one location per site, on the edge of plastic at Site 2 and the centre of mesh at Site 3; microhabitat colonization was the inner microhabitat on plastic at Site 2 and outer on mesh at Site 3. Porifera msp. 3 colonized just one location: centre of mesh at both Site 1 and 2. Broader microhabitat colonization occurred on mesh at Site 1 in both outer and middle microhabitats, while on mesh at Site 2 it colonized just the outer. A biological aggregate (Unknown msp. 3) was present on the edge of wood at Site 2; inner microhabitat colonization occurred on wood at Site 2 and it was present internally on mesh at Site 3.

The remaining seven mspp recruited to or colonized one or more locations and microhabitats but restricted to just one site (Figure 6C). This included two colonial hydrozoans colonizing locations and microhabitats broadly (Campanulariidae msp. 1 and *Eudendrium* msp. 2). Campanulariidae msp. 1 occurred in all locations and microhabitats available on stone at Site 1; *Eudendrium* msp. 2, similarly colonized all locations and microhabitats available on mesh at Site 4. Conversely, another colonial hydrozoan Hydrozoa msp. 1 occurred solely in centre location of stone at Site 1, colonizing the outer microhabitat alone. Foraminifera msp. 2 was present in the centre locations of mesh and stone at Site 4, colonizing the outer microhabitat on the former and the inner on the latter. Foraminifera msp. 4 occurred in the centre of plastic at Site 3, as well as being found internally on mesh at the same site. Its microhabitat colonization of the former occurred in the middle.

Supplementary tables

Table S1. Recruitment location on the substratum (see Figure 2 for details).

**Location**	**Description**
Center	Substratum area excluding Corner, Edge, and bolt holes*
Edge	Within 5 mm of sides
Corner	5 x 5 mm box including corner
Epibiota	Using another morphospecies as substratum

*Holes through the center of two opposite faces of the panels where they were bolted to the frame.

Table S2. Recruitment features or “microhabitats” on the substratum types (see Figure 2 for details)

Microhabitat	Description
**Mesh**	
Unsheltered	Outermost area
Sheltered	Below outermost surface, between and inside sheets of mesh
**Plastic**	
Unsheltered	Outermost area at top of plastic protuberances
Sheltered	Side or between plastic protuberances, and surface at base
**Stone**	
Unsheltered	Outermost area
Sheltered	Inside crevices and indentations below surface
**Wood**	
Unsheltered	Outermost area
Sheltered	Found below surface of substratum (boring)

Table S3. Deployment information for all settlement frames on moorings and landers.

Name	Site ID	Method	Depth (m)	Altitude (m)	Deployed	Recovered	Latitude	Longitude
Site 1	HiBioA	Mooring	499	10	Oct 2017	Jul 2018	60.46083° N	– 61.26217° W
Site 2	HiBioC	Mooring	960	60	Aug 2018	Jul 2019	60.46406° N	– 61.15908° W
Site 3	SpongeSite3	Lander	410	1	Jul 2018	Jul 2019	60.46738° N	– 61.28785° W
Site 4	HiBioA	Mooring	505	16	Jul 2019	Aug 2020	60.47417° N	– 60.26944° W
Site 5*	HiBioB	Mooring	1855	30	Aug 2018	-	60.47365° N	– 60.37526° W
Site 6*	HiBioC	Mooring	1025	12	Jul 2019	-	60.46405° N	– 61.15780° W

*Not recovered

Table S4. Total abundance of all individuals or colonies of all morphospecies present on four substratum types at four sites in the Labrador Sea (Canada).

Phylum/ Morphospecies	Site 1 Mesh	Plastic	Stone	Wood	*Site 1 Total*	Site 2 Mesh	Plastic	Stone	Wood	*Site 2 Total*	Site 3 Mesh	Plastic	Stone	Wood	*Site 3 Total*	Site 4 Mesh	Plastic	Stone	Wood	*Site 4 Total*	*Mesh Total*	*Plastic Total*	*Stone Total*	*Wood Total*	*All blocks pooled*
**Annelida**	0	0	0	0	0	0	0	0	0	0	2	0	0	0	2	0	0	1	0	1	2	0	1	0	3
Polychaeta mmsp. 1	0	0	0	0	0	0	0	0	0	0	1	0	0	0	1	0	0	1	0	1	1	0	1	0	2
Polychaeta mmsp. 2	0	0	0	0	0	0	0	0	0	0	1	0	0	0	1	0	0	0	0	0	1	0	0	0	1
**Arthropoda**	22	0	0	0	22	20	1	0	0	21	7	11	13	0	31	49	3	0	1	53	98	15	13	1	127
Caprellidae mmsp. 1	1	0	0	0	1	0	0	0	0	0	0	0	0	0	0	19	1	0	0	20	20	1	0	0	21
Copepoda mmsp. 1	16	0	0	0	16	20	1	0	0	21	2	1	0	0	3	20	2	0	1	23	58	4	0	1	63
Gammaridea mmsp. 1	0	0	0	0	0	0	0	0	0	0	5	10	13	0	28	0	0	0	0	0	5	10	13	0	28
Gammaridea mmsp. 2	4	0	0	0	4	0	0	0	0	0	0	0	0	0	0	0	0	0	0	0	4	0	0	0	4
Halacaridae msp. 1	1	0	0	0	1	0	0	0	0	0	0	0	0	0	0	0	0	0	0	0	1	0	0	0	1
Isopoda msp. 1	0	0	0	0	0	0	0	0	0	0	0	0	0	0	0	9	0	0	0	9	9	0	0	0	9
Ostracoda msp. 1	0	0	0	0	0	0	0	0	0	0	0	0	0	0	0	1	0	0	0	1	1	0	0	0	1
**Cnidaria**	24497	11296	20462	585	56841	154	12132	16	40	12342	93	4902	3732	0	8728	16130	13414	17212	2524	49281	40875	41745	41422	3149	127191
Actinaria msp. 1	1	0	0	0	1	0	0	0	0	0	0	0	0	0	0	2	1	0	0	3	3	1	0	0	4
Campanulariidae msp. 1	0	0	0	0	0	0	0	13	0	13	0	0	0	0	0	0	0	0	0	0	0	0	13	0	13
Campanulariidae msp. 2A	64	67	29	0	160	0	0	0	0	0	7	3	6	0	16	97	116	78	73	364	168	186	113	73	540
Campanulariidae msp. 2B	0	625	327	0	951	0	0	0	0	0	0	139	0	0	139	0	1076	897	850	2822	0	1840	1224	850	3913
Campanulariidae msp. 3	23696	10489	19793	585	54563	154	12132	3	40	12329	56	4043	2823	0	6922	9815	10683	15525	1510	37533	33721	37347	38145	2135	111347
Campanulariidae msp. 4	1	0	0	0	1	0	0	0	0	0	0	0	0	0	0	15	0	0	0	15	16	0	0	0	16
Eudendrium msp. 1	734	114	312	0	1160	0	0	0	0	0	30	717	903	0	1650	1802	1538	707	91	4138	2567	2368	1922	91	6948
Eudendrium msp. 2	0	0	0	0	0	0	0	0	0	0	0	0	0	0	0	4396	0	0	0	4396	4396	0	0	0	4396
Hydrozoa msp. 1	0	0	0	0	0	0	0	0	0	0	0	0	0	0	0	0	0	2	0	2	0	0	2	0	2
Octocorallia msp. 1	1	2	1	0	4	0	0	0	0	0	0	0	0	0	0	3	1	3	0	7	4	3	4	0	11
**Foraminifera**	134	0	2	1	137	6	0	0	0	6	88	2	1	0	91	10	0	5	0	15	238	2	8	1	249
Foraminifera msp. 1	134	0	2	1	137	5	0	0	0	5	87	1	1	0	89	7	0	4	0	11	233	1	7	1	242
Foraminifera msp. 2	0	0	0	0	0	0	0	0	0	0	0	0	0	0	0	1	0	1	0	2	1	0	1	0	2
Foraminifera msp. 3	0	0	0	0	0	1	0	0	0	1	0	0	0	0	0	2	0	0	0	2	3	0	0	0	3
Foraminifera msp. 4	0	0	0	0	0	0	0	0	0	0	1	1	0	0	2	0	0	0	0	0	1	1	0	0	2
**Mollusca**	0	0	0	0	0	0	0	0	0	0	0	0	0	0	0	35	0	0	0	35	35	0	0	0	35
Gastropoda msp. 1	0	0	0	0	0	0	0	0	0	0	0	0	0	0	0	1	0	0	0	1	1	0	0	0	1
Gastropoda msp. 2	0	0	0	0	0	0	0	0	0	0	0	0	0	0	0	34	0	0	0	34	34	0	0	0	34
**Porifera**	2	0	0	0	2	1	0	0	0	1	1	0	0	0	1	0	0	0	0	0	4	0	0	0	4
Porifera msp. 1	0	0	0	0	0	0	0	0	0	0	1	0	0	0	1	0	0	0	0	0	1	0	0	0	1
Porifera msp. 2	1	0	0	0	1	1	0	0	0	1	0	0	0	0	0	0	0	0	0	0	2	0	0	0	2
Porifera msp. 3	1	0	0	0	1	0	0	0	0	0	0	0	0	0	0	0	0	0	0	0	1	0	0	0	1
**Radiolaria**	0	0	0	0	0	1	0	1	0	2	2	0	0	0	2	2	1	1	0	4	5	1	2	0	8
Radiolaria msp. 1	0	0	0	0	0	1	0	1	0	2	2	0	0	0	2	2	1	1	0	4	5	1	2	0	8
**Unknown**	0	0	99	0	99	0	0	2	1	3	1	0	0	0	1	2	0	2	0	4	3	0	103	1	107
Unknown msp. 1	0	0	99	0	99	0	0	2	0	2	0	0	0	0	0	0	0	2	0	2	0	0	103	0	103
Unknown msp. 2	0	0	0	0	0	0	0	0	0	0	0	0	0	0	0	2	0	0	0	2	2	0	0	0	2
Unknown msp. 3	0	0	0	0	0	0	0	0	1	1	1	0	0	0	1	0	0	0	0	0	1	0	0	1	2
Species pooled	24655	11296	20563	586	57101	182	12133	19	41	12375	194	4915	3746	0	8856	16228	13418	17221	2525	49393	41260	41763	41549	3152	127724

Table S5. Density of all individuals or colonies per cm^2 of all morphospecies present on four substratum types at four sites in the Labrador Sea (Canada).

Phylum/ Species	Site 1 Mesh	Plastic	Stone	Wood	*Site 1 Total*	Site 2 Mesh	Plastic	Stone	Wood	*Site 2 Total*	Site 3 Mesh	Plastic	Stone	Wood	*Site 3 Total*	Site 4 Mesh	Plastic	Stone	Wood	*Site 4 Total*	*Mesh Total*	*Plastic Total*	*Stone Total*	*Wood Total*	*All blocks pooled*
**Annelida**	**0**	**0**	**0**	**0**	***0***	**0**	**0**	**0**	**0**	***0***	**0.002472**	**0**	**0**	**0**	***0.00063***	**0**	**0**	**0.001459**	**0**	***0.00073***	***0.00063***	***0***	***0.00073***	***0***	*0.0006625*
Polychaeta msp. 1	0	0	0	0	*0*	0	0	0	0	*0*	0.002593	0	0	0	*0.00065*	0	0	0.002917	0	*0.00073*	*0.00065*	*0*	*0.00073*	*0*	*0.0003444*
Polychaeta msp. 2	0	0	0	0	*0*	0	0	0	0	*0*	0.002351	0	0	0	*0.00059*	0	0	0	0	*0*	*0.00059*	*0*	*0*	*0*	*0.0001469*
**Arthropoda**	**0.004693**	**0**	**0**	**0**	***0.00563***	**0.003751**	**0.000504**	**0**	**0**	***0.00433***	**0.001829**	**0.002268**	**0.002575**	**0**	***0.00262***	**0.009338**	**0.001461**	**0**	**0.002461**	***0.00465***	***0.00562***	***0.00187***	***0.00317***	***0.00029***	*0.0039283*
Caprellidae msp. 1	0.002589	0	0	0	*0.00065*	0	0	0	0	*0*	0	0	0	0	*0*	0.023258	0.003341	0	0	*0.00665*	*0.00646*	*0.00084*	*0*	*0*	*0.0018243*
Copepoda msp. 1	0.020978	0	0	0	*0.00524*	0.026258	0.003531	0	0	*0.00745*	0.005185	0.003363	0	0	*0.00214*	0.017701	0.006886	0	0.017229	*0.01045*	*0.01753*	*0.00344*	*0*	*0.00431*	*0.0063207*
Gammaridea msp. 1	0	0	0	0	*0*	0	0	0	0	*0*	0.00762	0.012514	0.018022	0	*0.00954*	0	0	0	0	*0*	*0.0019*	*0.00313*	*0.00451*	*0*	*0.0023848*
Gammaridea msp. 2	0.006694	0	0	0	*0.00167*	0	0	0	0	*0*	0	0	0	0	*0*	0	0	0	0	*0*	*0.00167*	*0*	*0*	*0*	*0.0004184*
Halacaridae msp. 1	0.002589	0	0	0	*0.00065*	0	0	0	0	*0*	0	0	0	0	*0*	0	0	0	0	*0*	*0.00065*	*0*	*0*	*0*	*0.0001618*
Isopoda msp. 1	0	0	0	0	*0*	0	0	0	0	*0*	0	0	0	0	*0*	0.022034	0	0	0	*0.00551*	*0.00551*	*0*	*0*	*0*	*0.0013771*
Ostracoda msp. 1	0	0	0	0	*0*	0	0	0	0	*0*	0	0	0	0	*0*	0.002371	0	0	0	*0.00059*	*0.00059*	*0*	*0*	*0*	*0.0001482*
**Cnidaria**	**1.257467**	**1.701541**	**1.094506**	**0.245626**	***5.49231***	**0.02398**	**0.85651**	**0.001109**	**0.058737**	***1.30164***	**0.009506**	**0.3348**	**0.285714**	0	***0.78104***	**0.717661**	**1.123788**	**1.091242**	**2.055479**	***3.45369***	***2.36894***	***4.09272***	***2.57971***	***0.33644***	2.8078393
Actinaria msp. 1	0.002589	0	0	0	*0.00065*	0	0	0	0	*0*	0	0	0	0	*0*	0.004513	0.00338	0	0	*0.00197*	*0.00178*	*0.00085*	*0*	*0*	*0.0006552*
Campanulariidae msp. 1	0	0	0	0	*0*	0	0	0.008357	0	*0.00209*	0	0	0	0	*0*	0	0	0	0	*0*	*0*	*0*	*0.00209*	*0*	*0.0005223*
Campanulariidae msp. 2A	0.059277	0.150447	0.022595	0	*0.05808*	0	0	0	0	*0*	0.008916	0.010246	0.016547	0	*0.00893*	0.05048	0.065779	0.056696	0.314438	*0.12185*	*0.02967*	*0.05662*	*0.02396*	*0.07861*	*0.0472139*
Campanulariidae msp. 2B	0	3.786513	0.759296	0	*1.13645*	0	0	0	0	*0*	0	0.469585	0	0	*0.1174*	0	3.635953	2.635653	14.64507	*5.22917*	*0*	*1.97301*	*0.84874*	*3.66127*	*1.6207546*
Campanulariidae msp. 3	12.00276	12.72666	9.919103	2.456263	*9.27619*	0.239797	8.565101	0.002734	0.587372	*2.34875*	0.065495	2.364879	2.309856	0	*1.18506*	4.268253	6.65449	7.781203	5.203308	*5.97681*	*4.14408*	*7.57778*	*5.00322*	*2.06174*	*4.6967043*
Campanulariidae msp. 4	0.002426	0	0	0	*0.00061*	0	0	0	0	*0*	0	0	0	0	*0*	0.011283	0	0	0	*0.00282*	*0.00343*	*0*	*0*	*0*	*0.0008568*
Eudendrium msp. 1	0.505194	0.339527	0.241741	0	*0.27162*	0	0	0	0	*0*	0.020647	0.503288	0.530738	0	*0.26367*	1.102654	0.874899	0.427002	0.391971	*0.69913*	*0.40712*	*0.42943*	*0.29987*	*0.09799*	*0.3086038*
Eudendrium msp. 2	0	0	0	0	*0*	0	0	0	0	*0*	0	0	0	0	*0*	1.736979	0	0	0	*0.43424*	*0.43424*	*0*	*0*	*0*	*0.1085612*
Hydrozoa msp. 1	0	0	0	0	*0*	0	0	0	0	*0*	0	0	0	0	*0*	0	0	0.005835	0	*0.00146*	*0*	*0*	*0.00146*	*0*	*0.0003647*
Octocorallia msp. 1	0.002426	0.012264	0.002323	0	*0.00425*	0	0	0	0	*0*	0	0	0	0	*0*	0.002448	0.00338	0.006028	0	*0.00296*	*0.00122*	*0.00391*	*0.00209*	*0*	*0.0018044*
**Foraminifera**	**0.015812**	**0**	**0.001157**	**0.005248**	***0.02808***	**0.003475**	**0**	**0**	**0**	***0.00145***	**0.009836**	**0.001784**	**0.000679**	0	***0.01058***	**0.003251**	**0**	**0.002258**	**0**	***0.00161***	***0.01648***	***0.00089***	***0.00126***	***0.00045***	*0.0099878*
Foraminifera msp. 1	0.063249	0	0.004626	0.020994	*0.02222*	0.011584	0	0	0	*0.0029*	0.036669	0.003521	0.002715	0	*0.01073*	0.006043	0	0.005921	0	*0.00299*	*0.02939*	*0.00088*	*0.00332*	*0.00525*	*0.0097076*
Foraminifera msp. 2	0	0	0	0	*0*	0	0	0	0	*0*	0	0	0	0	*0*	0.002257	0	0.00311	0	*0.00134*	*0.00056*	*0*	*0.00078*	*0*	*0.0003354*
Foraminifera msp. 3	0	0	0	0	*0*	0.002317	0	0	0	*0.00058*	0	0	0	0	*0*	0.004705	0	0	0	*0.00118*	*0.00176*	*0*	*0*	*0*	*0.0004389*
Foraminifera msp. 4	0	0	0	0	*0*	0	0	0	0	*0*	0.002676	0.003617	0	0	*0.00157*	0	0	0	0	*0*	*0.00067*	*0.0009*	*0*	*0*	*0.0003933*
**Mollusca**	**0**	**0**	**0**	**0**	***0***	**0**	**0**	**0**	**0**	***0***	**0**	**0**	**0**	0	***0***	**0.041428**	**0**	**0**	**0**	***0.01002***	***0.01002***	***0***	***0***	***0***	*0.0100226*
Gastropoda msp. 1	0	0	0	0	*0*	0	0	0	0	*0*	0	0	0	0	*0*	0.002257	0	0	0	*0.00056*	*0.00056*	*0*	*0*	*0*	*0.000141*
Gastropoda msp. 2	0	0	0	0	*0*	0	0	0	0	*0*	0	0	0	0	*0*	0.080599	0	0	0	*0.02015*	*0.02015*	*0*	*0*	*0*	*0.0050374*
**Porifera**	**0.001824**	**0**	**0**	**0**	***0.00164***	**0.000772**	**0**	**0**	**0**	***0.00088***	**0.000892**	**0**	**0**	0	***0.00082***	**0**	**0**	**0**	**0**	***0***	***0.00111***	***0***	***0***	***0***	*0.0011128*
Porifera msp. 1	0	0	0	0	*0*	0	0	0	0	*0*	0.002676	0	0	0	*0.00067*	0	0	0	0	*0*	*0.00067*	*0*	*0*	*0*	*0.0001673*
Porifera msp. 2	0.002737	0	0	0	*0.00068*	0.002317	0	0	0	*0.00058*	0	0	0	0	*0*	0	0	0	0	*0*	*0.00126*	*0*	*0*	*0*	*0.0003158*
Porifera msp. 3	0.002737	0	0	0	*0.00068*	0	0	0	0	*0*	0	0	0	0	*0*	0	0	0	0	*0*	*0.00068*	*0*	*0*	*0*	*0.000171*
**Radiolaria**	**0**	**0**	**0**	**0**	***0***	**0.002317**	**0**	**0.002804**	**0**	***0.00087***	**0.005269**	**0**	**0**	0	***0.00074***	**0.004513**	**0.00338**	**0.00311**	**0**	***0.00092***	***0.00083***	***0.00085***	***0.00092***	***0***	*0.0008546*
Radiozoa msp. 1	0	0	0	0	*0*	0.002317	0	0.002804	0	*0.00128*	0.005269	0	0	0	*0.00132*	0.004513	0.00338	0.00311	0	*0.00275*	*0.00302*	*0.00085*	*0.00148*	*0*	*0.0013371*
**Unknown**	**0**	**0**	**0.038944**	**0**	***0.04281***	**0**	**0**	**0.001869**	**0.004895**	***0.00136***	**0.000892**	**0**	**0**	0	***0.00082***	**0.001504**	**0**	**0.000972**	**0**	***0.0013***	***0.00098***	***0***	***0.02229***	***0.00062***	*0.0131071*
Unknown msp. 1	0	0	0.116833	0	*0.02921*	0	0	0.005607	0	*0.0014*	0	0	0	0	*0*	0	0	0.002917	0	*0.00073*	*0*	*0*	*0.03134*	*0*	*0.0078349*
Unknown msp. 2	0	0	0	0	*0*	0	0	0	0	*0*	0	0	0	0	*0*	0.004513	0	0	0	*0.00113*	*0.00113*	*0*	*0*	*0*	*0.0002821*
Unknown msp. 3	0	0	0	0	*0*	0	0	0	0.014684	*0.00367*	0.002676	0	0	0	*0.00067*	0	0	0	0	*0*	*0.00067*	*0*	*0*	*0.00367*	*0.001085*
Species pooled	*65.5377*	*85.9198*	*55.1222*	*1.9028*	*52.1206*	*1.42423*	*42.8486*	*0.09775*	*0.50575*	*11.2191*	*0.81089*	*16.7915*	*14.2945*	*0*	*10.6323*	*36.3966*	*56.5126*	*54.9234*	*12.0201*	*39.9632*	*26.0423*	*50.5182*	*31.7337*	*4.80956*	*28.4838*

Table S6. Surface and canopy coverage by all non-hydrozoan morphospecies and phyla across substratum types (mesh, plastic, stone, wood) and geographic sites (1, 2, 3, 4). A * indicates that the Cnidaria cover includes hydrozoans (see Figure 6 for detail).

Phylum/ Morphospecies	Surface cover (%)									Canopy cover (%)								
		Substratum type				Geographic location					Substratum type				Geographic location			
	Overall	Mesh	Plastic	Stone	Wood	Site 1	Site 2	Site 3	Site 4	*Overall*	Mesh	Plastic	Stone	Wood	Site 1	Site 2	Site 3	Site 4
**Annelida**	***3.0***	**3.7**	**0.0**	**1.0**	**0.0**	**5.0**	**0.0**	**3.0**	**1.0**	***0.0***	**0.0**	**0.0**	**0.0**	**0.0**	**0.0**	**0.0**	**0.0**	**0.0**
Polychaeta msp. 1	*3.0*	5.0	0.0	1.0	0.0	0.0	0.0	5.0	1.0	*0.0*	0.0	0.0	0.0	0.0	0.0	0.0	0.0	0.0
Polychaeta msp. 2	*3.0*	3.0	0.0	0.0	0.0	5.0	0.0	1.0	0.0	*0.0*	0.0	0.0	0.0	0.0	0.0	0.0	0.0	0.0
**Arthropoda**	***1.7***	**2.2**	**0.9**	**1.0**	**1.0**	**2.1**	**1.8**	**1.0**	**2.4**	***0.5***	**0.6**	**0.4**	**0.5**	**0.0**	**0.5**	**0.0**	**0.1**	**1.3**
Caprellidae msp. 1	*1.3*	1.7	0.0	0.0	0.0	0.0	0.0	0.0	1.7	*5.0*	5.0	5.0	0.0	0.0	5.0	0.0	0.0	5.0
Copepoda msp. 1	*2.1*	2.4	1.0	0.0	1.0	1.8	1.8	1.0	2.5	*0.0*	0.0	0.0	0.0	0.0	0.0	0.0	0.0	0.0
Gammaridea msp. 1	*1.0*	1.0	1.0	1.0	0.0	0.0	0.0	1.0	0.0	*0.1*	0.0	0.0	0.5	0.0	0.0	0.0	0.1	0.0
Gammaridea msp. 2	*3.7*	3.7	0.0	0.0	0.0	3.7	0.0	0.0	0.0	*0.0*	0.0	0.0	0.0	0.0	0.0	0.0	0.0	0.0
Halacaridae msp. 1	*1.0*	1.0	0.0	1.0	0.0	1.0	0.0	0.0	0.0	*0.0*	0.0	0.0	0.0	0.0	0.0	0.0	0.0	0.0
Isopoda msp. 1	*5.0*	5.0	0.0	0.0	0.0	0.0	0.0	0.0	5.0	*0.0*	0.0	0.0	0.0	0.0	0.0	0.0	0.0	0.0
Ostracoda msp. 1	*1.0*	1.0	0.0	0.0	0.0	0.0	0.0	0.0	1.0	*0.0*	0.0	0.0	0.0	0.0	0.0	0.0	0.0	0.0
**Cnidaria***	***11.7***	**8.2**	**11.8**	**10.8**	**37.1**	**13.6**	**9.0**	**6.4**	**13.7**	***11.1***	**10.1**	**10.5**	**9.1**	**32.1**	**11.2**	**2.8**	**5.9**	**15.8**
Actiniaria msp. 1	*1.0*	1.0	1.0	1.0	0.0	1.0	0.0	0.0	1.0	*0.3*	1.0	0.0	0.0	0.0	0.0	0.0	0.0	0.5
Octocorallia msp. 1	*1.0*	1.0	1.0	1.0	0.0	1.0	0.0	0.0	1.0	*2.6*	0.0	0.0	2.6	0.0	0.0	2.6	0.0	0.0
**Foraminifera**	***1.4***	**1.5**	**1.0**	**0.9**	**1.0**	**3.2**	**1.0**	**1.0**	**0.8**	***11.4***	**9.2**	**12.5**	**8.5**	**32.5**	**8.9**	**0.0**	**2.3**	**14.9**
Foraminifera msp. 1	*1.4*	1.5	1.0	0.8	1.0	3.2	1.0	1.0	0.7	*10.3*	6.6	5.9	12.8	42.7	15.3	2.9	5.3	12.1
Foraminifera msp. 2	*1.0*	1.0	0.0	1.0	0.0	0.0	0.0	0.0	1.0	*3.4*	3.4	0.0	0.0	0.0	1.0	0.0	0.0	5.0
Foraminifera msp. 3	*1.0*	1.0	0.0	0.0	0.0	0.0	1.0	0.0	1.0	*12.2*	5.5	21.0	9.7	10.3	7.5	0.0	7.7	18.5
Foraminifera msp. 4	*1.0*	0.0	1.0	0.0	0.0	0.0	0.0	1.0	0.0	*72.5*	72.5	0.0	0.0	0.0	0.0	0.0	0.0	72.5
**Mollusca**	***0.5***	**0.5**	**0.0**	**0.0**	**0.0**	**0.0**	**0.0**	**0.0**	**0.5**	***1.0***	**0.0**	**0.0**	**1.0**	**0.0**	**0.0**	**0.0**	**0.0**	**1.0**
Gastropoda msp. 1	*1.0*	1.0	0.0	0.0	0.0	0.0	0.0	0.0	1.0	*0.0*	0.0	0.0	0.0	0.0	0.0	0.0	0.0	0.0
Gastropoda msp. 2	*0.0*	0.0	0.0	0.0	0.0	0.0	0.0	0.0	0.0	*6.7*	0.0	8.0	6.7	0.0	10.0	0.0	2.5	6.3
**Porifera**	***2.0***	**2.3**	**1.0**	**0.0**	**0.0**	**1.0**	**1.0**	**5.0**	**0.0**	***0.1***	**0.1**	**0.0**	**0.1**	**0.0**	**0.1**	**0.0**	**0.0**	**0.2**
Porifera msp. 1	*3.0*	5.0	1.0	0.0	0.0	0.0	1.0	5.0	0.0	*0.1*	0.1	0.0	0.2	0.0	0.1	0.0	0.0	0.3
Porifera msp. 2	*1.0*	1.0	0.0	0.0	0.0	1.0	1.0	0.0	0.0	*0.0*	0.0	0.0	0.0	0.0	0.0	0.0	0.0	0.0
Porifera msp. 3	*0.0*	0.0	0.0	0.0	0.0	0.0	0.0	0.0	0.0	*0.0*	0.0	0.0	0.0	0.0	0.0	0.0	0.0	0.0
**Radiolaria**	***1.0***	**1.0**	**1.0**	**1.0**	**0.0**	**0.0**	**1.0**	**0.0**	**1.0**	***0.0***	**0.0**	**0.0**	**0.0**	**0.0**	**0.0**	**0.0**	**0.0**	**0.0**
Radiolaria msp. 1	*1.0*	1.0	1.0	1.0	0.0	0.0	1.0	0.0	1.0	*2.5*	2.5	0.0	0.0	0.0	0.0	0.0	0.0	2.5
**Unknown**	***1.9***	**0.0**	**1.0**	**2.2**	**1.0**	**3.0**	**1.0**	**0.0**	**0.5**	***0.0***	**0.0**	**0.0**	**0.0**	**0.0**	**0.0**	**0.0**	**0.0**	**0.0**
Unknown msp. 1	*2.0*	0.0	1.0	2.2	0.0	3.0	1.0	0.0	0.5	*5.0*	5.0	0.0	0.0	0.0	0.0	0.0	0.0	5.0
Unknown msp. 2	*0.0*	0.0	0.0	0.0	0.0	0.0	0.0	0.0	0.0	*0.0*	0.0	0.0	0.0	0.0	0.0	0.0	0.0	0.0
Unknown msp. 3	*1.0*	0.0	0.0	0.0	1.0	0.0	1.0	0.0	0.0	*0.0*	0.0	0.0	0.0	0.0	0.0	0.0	0.0	0.0

Table S7. Base coverage (%) of all individuals or colonies of all morphospecies present on four substratum types at four sites in the Labrador Sea (Canada).

	Site 1				Site 2				Site 3			Site 4			
**Species**	**Mesh**	**Plastic**	**Stone**	**Wood**	**Mesh**	**Plastic**	**Stone**	**Wood**	**Mesh**	**Plastic**	**Stone**	**Mesh**	**Plastic**	**Stone**	**Wood**
**Annelida**	**0**	**0**	**0**	**0**	**0**	**0**	**0**	**0**	**6**	**0**	**0**	**0**	**0**	**1**	**0**
Polychaeta msp. 1	0	0	0	0	0	0	0	0	5	0	0	0	0	1	0
Polychaeta msp. 2	0	0	0	0	0	0	0	0	1	0	0	0	0	0	0
**Arthropoda**	**6**	**0**	**0**	**0**	**2**	**1**	**0**	**0**	**1**	**2**	**1**	**12**	**1**	**0**	**1**
Caprellidae msp. 1	0	0	0	0	0	0	0	0	0	0	0	3	0	0	0
Copepoda msp. 1	1	0	0	0	2	1	0	0	0	1	0	3	1	0	1
Gammaridea msp. 1	0	0	0	0	0	0	0	0	1	1	1	0	0	0	0
Gammaridea msp. 2	4	0	0	0	0	0	0	0	0	0	0	0	0	0	0
Halacaridae msp. 1	1	0	0	0	0	0	0	0	0	0	0	0	0	0	0
Isopoda msp. 1	0	0	0	0	0	0	0	0	0	0	0	5	0	0	0
Ostracoda msp. 1	0	0	0	0	0	0	0	0	0	0	0	1	0	0	0
**Cnidaria**	**23**	**48**	**36**	**62**	**9**	**13**	**15**	**5**	**5**	**13**	**32**	**82**	**47**	**38**	**131**
Actinaria msp. 1	0	0	0	0	0	0	0	0	0	0	0	1	1	0	0
Campanulariidae msp. 1	0	0	0	0	0	0	5	0	0	0	0	0	0	0	0
Campanulariidae msp. 2A	4	8	3	0	0	0	0	0	1	1	5	4	5	6	17
Campanulariidae msp. 2B	0	8	5	0	0	0	0	0	0	3	0	0	5	5	50
Campanulariidae msp. 3	11	28	18	62	9	13	10	5	2	6	18	8	24	18	52
Campanulariidae msp. 4	1	0	0	0	0	0	0	0	0	0	0	1	0	0	0
Eudendrium msp. 1	6	3	8	0	0	0	0	0	2	3	10	5	11	8	13
Eudendrium msp. 2	0	0	0	0	0	0	0	0	0	0	0	62	0	0	0
Hydrozoa msp. 1	0	0	0	0	0	0	0	0	0	0	0	0	0	1	0
Octocorallia msp. 1	1	1	1	0	0	0	0	0	0	0	0	1	1	1	0
**Foraminifera**	**4**	**0**	**1**	**1**	**2**	**0**	**0**	**0**	**1**	**2**	**1**	**3**	**0**	**2**	**0**
Foraminifera msp. 1	4	0	1	1	1	0	0	0	1	1	1	1	0	1	0
Foraminifera msp. 2	0	0	0	0	0	0	0	0	0	0	0	1	0	1	0
Foraminifera msp. 3	0	0	0	0	1	0	0	0	0	0	0	1	0	0	0
Foraminifera msp. 4	0	0	0	0	0	0	0	0	0	1	0	0	0	0	0
**Mollusca**	**0**	**0**	**0**	**0**	**0**	**0**	**0**	**0**	**0**	**0**	**0**	**1**	**0**	**0**	**0**
Gastropoda msp. 1	0	0	0	0	0	0	0	0	0	0	0	1	0	0	0
Gastropoda msp. 2	0	0	0	0	0	0	0	0	0	0	0	0	0	0	0
**Porifera**	**1**	**0**	**0**	**0**	**1**	**0**	**0**	**0**	**5**	**0**	**0**	**0**	**0**	**0**	**0**
Porifera msp. 1	0	0	0	0	0	0	0	0	5	0	0	0	0	0	0
Porifera msp. 2	1	0	0	0	1	0	0	0	0	0	0	0	0	0	0
Porifera msp. 3	0	0	0	0	0	0	0	0	0	0	0	0	0	0	0
**Radiolaria**	**0**	**0**	**0**	**0**	**1**	**0**	**1**	**0**	**0**	**0**	**0**	**0**	**1**	**1**	**0**
Radiolaria msp. 1	0	0	0	0	1	0	1	0	0	0	0	0	1	1	0
**Unknown**	**0**	**0**	**4**	**0**	**0**	**0**	**1**	**1**	**0**	**0**	**0**	**0**	**0**	**1**	**0**
Unknown msp. 1	0	0	4	0	0	0	1	0	0	0	0	0	0	1	0
Unknown msp. 2	0	0	0	0	0	0	0	0	0	0	0	0	0	0	0
Unknown msp. 3	0	0	0	0	0	0	0	1	0	0	0	0	0	0	0

Table S8. Canopy coverage (%) of all individuals or colonies of all morphospecies present on four substratum types at four sites in the Labrador Sea (Canada).

	Site 1				Site 2				Site 3			Site 4			
**Species**	**Mesh**	**Plastic**	**Stone**	**Wood**	**Mesh**	**Plastic**	**Stone**	**Wood**	**Mesh**	**Plastic**	**Stone**	**Mesh**	**Plastic**	**Stone**	**Wood**
**Annelida**	**0**	**0**	**0**	**0**	**0**	**0**	**0**	**0**	**0**	**0**	**0**	**0**	**0**	**0**	**0**
Polychaeta msp. 1	0	0	0	0	0	0	0	0	0	0	0	0	0	0	0
Polychaeta msp. 2	0	0	0	0	0	0	0	0	0	0	0	0	0	0	0
**Arthropoda**	**5**	**0**	**0**	**0**	**0**	**0**	**0**	**0**	**0**	**0**	**1**	**5**	**5**	**0**	**0**
Caprellidae msp. 1	5	0	0	0	0	0	0	0	0	0	0	5	5	0	0
Copepoda msp. 1	0	0	0	0	0	0	0	0	0	0	0	0	0	0	0
Gammaridea msp. 1	0	0	0	0	0	0	0	0	0	0	1	0	0	0	0
Gammaridea msp. 2	0	0	0	0	0	0	0	0	0	0	0	0	0	0	0
Halacaridae msp. 1	0	0	0	0	0	0	0	0	0	0	0	0	0	0	0
Isopoda msp. 1	0	0	0	0	0	0	0	0	0	0	0	0	0	0	0
Ostracoda msp. 1	0	0	0	0	0	0	0	0	0	0	0	0	0	0	0
**Cnidaria**	**24**	**62**	**29**	**53**	**6**	**2**	**9**	**5**	**5**	**15**	**31**	**108**	**61**	**40**	**83**
Actinaria msp. 1	0	0	0	0	0	0	0	0	0	0	0	1	0	0	0
Campanulariidae msp. 1	0	0	0	0	0	0	2	0	0	0	0	0	0	0	0
Campanulariidae msp. 2A	7	19	3	0	0	0	0	0	1	5	5	16	11	13	33
Campanulariidae msp. 2B	0	13	5	0	0	0	0	0	0	3	0	0	10	8	0
Campanulariidae msp. 3	9	13	15	53	6	2	7	5	2	2	14	6	8	12	40
Campanulariidae msp. 4	1	0	0	0	0	0	0	0	0	0	0	5	0	0	0
Eudendrium msp. 1	7	18	5	0	0	0	0	0	2	6	13	7	31	7	10
Eudendrium msp. 2	0	0	0	0	0	0	0	0	0	0	0	73	0	0	0
Hydrozoa msp. 1	0	0	0	0	0	0	0	0	0	0	0	0	0	1	0
Octocorallia msp. 1	0	0	0	0	0	0	0	0	0	0	0	0	0	0	0
**Foraminifera**	**0**	**0**	**0**	**0**	**0**	**0**	**0**	**0**	**0**	**0**	**0**	**0**	**0**	**0**	**0**
Foraminifera msp. 1	0	0	0	0	0	0	0	0	0	0	0	0	0	0	0
Foraminifera msp. 2	0	0	0	0	0	0	0	0	0	0	0	0	0	0	0
Foraminifera msp. 3	0	0	0	0	0	0	0	0	0	0	0	0	0	0	0
Foraminifera msp. 4	0	0	0	0	0	0	0	0	0	0	0	0	0	0	0
**Mollusca**	**0**	**0**	**0**	**0**	**0**	**0**	**0**	**0**	**0**	**0**	**0**	**5**	**0**	**0**	**0**
Gastropoda msp. 1	0	0	0	0	0	0	0	0	0	0	0	0	0	0	0
Gastropoda msp. 2	0	0	0	0	0	0	0	0	0	0	0	5	0	0	0
**Porifera**	**0**	**0**	**0**	**0**	**0**	**0**	**0**	**0**	**0**	**0**	**0**	**0**	**0**	**0**	**0**
Porifera msp. 1	0	0	0	0	0	0	0	0	0	0	0	0	0	0	0
Porifera msp. 2	0	0	0	0	0	0	0	0	0	0	0	0	0	0	0
Porifera msp. 3	0	0	0	0	0	0	0	0	0	0	0	0	0	0	0
**Radiolaria**	**0**	**0**	**0**	**0**	**0**	**0**	**0**	**0**	**0**	**0**	**0**	**0**	**0**	**0**	**0**
Radiolaria msp. 1	0	0	0	0	0	0	0	0	0	0	0	0	0	0	0
**Unknown**	**0**	**0**	**0**	**0**	**0**	**0**	**0**	**0**	**0**	**0**	**0**	**0**	**0**	**1**	**0**
Unknown msp. 1	0	0	0	0	0	0	0	0	0	0	0	0	0	1	0
Unknown msp. 2	0	0	0	0	0	0	0	0	0	0	0	0	0	0	0
Unknown msp. 3	0	0	0	0	0	0	0	0	0	0	0	0	0	0	0

Table S9. Results of PERMANOVA and two-way crossed analysis of similarity (ANOSIM) tests on Bray-Curtis resemblance matrices on the four substratum types and four geographic sites in Labrador Sea (Canada). Global results are indicated in bold.

	PERMANOVA		ANOSIM	
Substratum Type	Pseudo-F	Significance (p)	Dissimilarity (ρ)	Significance (p)
**Base coverage**	**5.28**	**<0.001**	**0.441**	**<0.001**
Mesh, Plastic			0.583	<0.001
Mesh, Stone			0.556	0.004
Mesh, Wood			0.704	0.047
Plastic, Stone			0.213	0.067
Plastic, Wood			0.852	0.016
Stone, Wood			0.259	0.172
**Canopy coverage**	**3.39**	**<0.001**	**0.352**	**<0.001**
Mesh, Plastic			0.37	0.005
Mesh, Stone			0.463	0.002
Mesh, Wood			0.111	0.422
Plastic, Stone			0.389	0.006
Plastic, Wood			0.704	0.016
Stone, Wood			0.333	0.125
**Abundance**	**8.15**	**<0.001**	**0.471**	**<0.001**
Mesh, Plastic			0.657	<0.001
Mesh, Stone			0.593	0.001
Mesh, Wood			0.63	0.063
Plastic, Stone			0.185	0.125
Plastic, Wood			0.704	0.047
Stone, Wood			0.556	0.109
**Density**	**6.74**	**<0.001**	**0.405**	**<0.001**
Mesh, Plastic			0.5	0.006
Mesh, Stone			0.463	0.006
Mesh, Wood			0.556	0.078
Plastic, Stone			0.157	0.163
Plastic, Wood			0.704	0.047
Stone, Wood			0.407	0.188
**Shannon diversity**	**1.07**	**0.374**	**0.212**	**0.025**
Mesh, Plastic			0.339	0.019
Mesh, Stone			0.139	0.172
Mesh, Wood			0.556	0.078
Plastic, Stone			0.021	0.374
Plastic, Wood			0.407	0.094
Stone, Wood			0.185	0.266
**Species richness**	**8.62**	**<0.001**	**0.326**	**0.005**
Mesh, Plastic			0.569	0.001
Mesh, Stone			0.514	<0.001
Mesh, Wood			1	0.016
Plastic, Stone			-0.046	0.58
Plastic, Wood			0.037	0.484
Stone, Wood			-0.296	1
**Phylum richness**	**9.19**	**<0.001**	**0.247**	**0.019**
Mesh, Plastic			0.625	<0.001
Mesh, Stone			0.366	0.01
Mesh, Wood			0.852	0.031
Plastic, Stone			-0.13	0.761
Plastic, Wood			-0.222	1
Stone, Wood			-0.333	0.969

	PERMANOVA		ANOSIM	
Geographic site	Pseudo-F	Significance (p)	Dissimilarity (ρ)	Significance (p)
**Base coverage**	**10.05**	**<0.001**	**0.67**	**<0.001**
Site 1, Site 2			0.951	0.001
Site 1, Site 3			0.58	0.006
Site 1, Site 4			0.025	0.405
Site 2, Site 3			0.901	0.001
Site 2, Site 4			0.84	0.001
Site 3, Site 4			0.63	0.002
**Canopy coverage**	**10.11**	**<0.001**	**0.592**	**<0.001**
Site 1, Site 2			0.938	0.001
Site 1, Site 3			0.383	0.032
Site 1, Site 4			0.012	0.456
Site 2, Site 3			0.704	0.003
Site 2, Site 4			0.963	0.001
Site 3, Site 4			0.494	0.003
**Abundance**	**10.99**	**<0.001**	**0.617**	**<0.001**
Site 1, Site 2			0.889	0.001
Site 1, Site 3			0.753	0.002
Site 1, Site 4			-0.062	0.599
Site 2, Site 3			0.926	0.001
Site 2, Site 4			0.827	0.001
Site 3, Site 4			0.457	0.002
**Density**	**9.31**	**<0.001**	**0.553**	**<0.001**
Site 1, Site 2			0.877	0.001
Site 1, Site 3			0.778	0.001
Site 1, Site 4			-0.025	0.49
Site 2, Site 3			0.901	0.001
Site 2, Site 4			0.605	0.002
Site 3, Site 4			0.259	0.034
**Shannon diversity**	**3.82**	**<0.001**	**0.292**	**<0.001**
Site 1, Site 2			0.267	0.033
Site 1, Site 3			0.346	0.01
Site 1, Site 4			0.074	0.324
Site 2, Site 3			0.558	0.001
Site 2, Site 4			0.304	0.04
Site 3, Site 4			0.259	0.077
**Species richness**	**9.87**	**<0.001**	**0.328**	**0.002**
Site 1, Site 2			0.543	0.01
Site 1, Site 3			0.006	0.436
Site 1, Site 4			0.08	0.814
Site 2, Site 3			0.58	0.004
Site 2, Site 4			0.765	0.001
Site 3, Site 4			0.012	0.507
**Phylum richness**	**2.51**	**0.07**	**0.066**	**0.237**
Site 1, Site 2			-0.167	1
Site 1, Site 3			0.296	0.056
Site 1, Site 4			0.056	0.472
Site 2, Site 3			0.222	0.13
Site 2, Site 4			0.111	0.262
Site 3, Site 4			-0.117	0.73

	PERMANOVA	
Site x Substratum type	Pseudo-F	Significance (p)
**Base coverage**	**3.01**	**<0.001**
**Canopy coverage**	3.01	<0.001
**Abundance**	3.46	<0.001
**Density**	3.64	<0.001
**Shannon diversity**	2.35	0.002
**Species richness**	0.89	0.549
**Phylum richness**	0.55	0.82

Supplementary figures

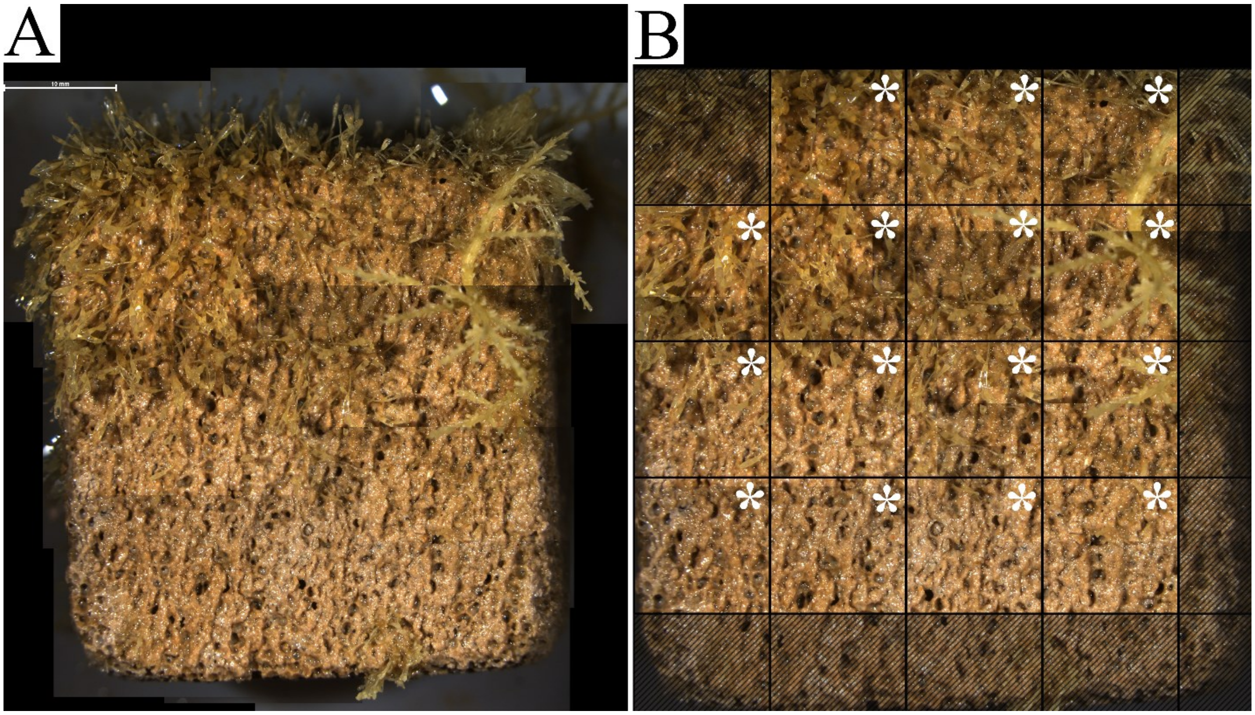

Figure S1. Sample of a mosaic image and the grid overlays used for morphospecies abundance measurements from “Face #2” of “Stone #1” of Site 4, e.g., the second face examined on one of the three carbonate block replicates at Site 4. Cross-hatching indicates squares excluded on all substratum types due to erosion (corners), or an incomplete grid square. Scale bar is 10 mm. (A) The mosaic image generated using individual photographs taken of each section of the surface using the Leica M205 stereo microscope and LAS-X software, then stitched together in Adobe Photoshop CS6. (B) A grid overlay of 1 cm squares on the mosaic image, used for surfaces with more sparse colonization.
